# Analysis of ESAC-Net/EARS-Net Data from 29 EEA Countries for Spatiotemporal Associations Between Antimicrobial Use and Resistance—Implications for Antimicrobial Stewardship?

**DOI:** 10.3390/antibiotics14040399

**Published:** 2025-04-13

**Authors:** James C. McSorley

**Affiliations:** Department of Microbiology, Level 4, New Lister Building, Glasgow Royal Infirmary, Alexandra Parade, Glasgow, Scotland G31 2ER, UK; james.mcsorley3@nhs.scot

**Keywords:** antimicrobial resistance, stewardship, EARS-NET, ESAC-NET, ESBL, MRSA, VRE, AWaRe

## Abstract

Background/Objectives: Antimicrobial resistance is one of the foremost global health concerns of today, and it could offset much of the progress accrued in healthcare over the last century. Excessive antibiotic use accelerates this problem, but it is recognised that specific agents differ in their capacity to promote resistance, a concept recently promoted by the World Health Organisation in the form of its Access, Watch, Reserve (AWaRe) schema. Which, if any, agents should be construed as having a high proclivity for selection of resistance has been contested. The European Antimicrobial Resistance Surveillance Network (EARS-NET) and European Surveillance of Antimicrobial Consumption Network (ESAC-NET) curate population level data over time and throughout the European Economic Area (EEA). EARS-NET monitors resistance to antimicrobials amongst invasive isolates of sentinel pathogens whereas ESAC-NET tracks usage of systemic antimicrobials. Together, data from these networks were interrogated to delineate correlations between antimicrobial consumption and resistance. Methods: Using univariate and multivariate regression analyses, spatiotemporal associations between the use of specific antimicrobial classes and 14 key resistance phenotypes in five sentinel pathogens were assessed methodically for 29 EEA countries. Results: Use of second and third generation cephalosporins, extended spectrum penicillin/β-lactamase inhibitor combinations, carbapenems, fluoroquinolones, nitroimidazoles and macrolides strongly correlated with key resistance phenotypes, as did overall antimicrobial consumption. Conclusions: The data obtained mostly support the WHO AWaRe schema with critical caveats. They have the potential to inform antimicrobial stewardship initiatives in the EEA, highlighting obstacles and shortcomings which may be modified in future to minimise positive selection for problematic resistance.

## 1. Introduction

It is forecast that AMR will account for 10 million deaths per annum by 2050 unless countered by radical action [1]. Improvident use of antimicrobials is liable to hasten the selection of resistant pathogens, but it is recognised that some agents have greater ecological impact than others [1,2,3,4,5,6]. Drugs with a proclivity to quickly select for resistance after even limited use can be seen as having a high resistance potential [6,7]. Conversely, agents which select for resistance only after heavy use can be considered as having a low resistance potential [6,7]. Emphatically, this concept is a gross generalisation with critical exceptions. No drug thus far has been ‘resistance-proof’, and resistance will inevitably emerge if consumption is sustained beyond a certain threshold [8,9,10,11]. Lavish use of any antibiotic, no matter how low its perceived resistance potential, should therefore be discouraged. Pathogens vary in their capacity to acquire resistance to specific agents [6,7,8,9,10]. We are only now, ca. 80 years after the clinical debut of penicillin, seeing the first signs of resistance developing in *Streptococcus pyogenes* [12,13]. A distinct scenario occurred with *Staphylococcus aureus*, penicillin resistant strains of which emerged and spread swiftly, gaining dominance first in hospitals and then the wider community [14,15,16]. Regarding the rapidity with which penicillin resistance was acquired, most other inherently sensitive pathogens including pneumococci, gonococci and meningococci fell between these superlatives but, overall, resistance emerged only gradually after decades of intense use [17,18,19]. Penicillin could therefore be viewed as possessing a low resistance potential. Contrastingly, third generation cephalosporins were quickly met with resistance problems in the form of nosocomial outbreaks due to multi-resistant Gram-negative bacilli (MDR-GNB) [20,21,22,23,24,25,26]. These agents also became associated with methicillin resistant *S. aureus* (MRSA) and vancomycin resistant enterococci (VRE), more so than did the antistaphylococcal penicillins and vancomycin themselves, respectively [26,27,28,29,30,31,32,33]. Resistance to the prototype fluoroquinolone ciprofloxacin by epidemic MRSA (EMRSA-15) clone ST22-A2, arose rapidly after its introduction in the UK, and was a pivotal factor in its successful pandemic spread [34]. Moreover, third generation cephalosporins and fluoroquinolones each pose distinctly high risks of promoting *Clostridioides difficile* infection [26,35,36,37,38,39]. These drugs could therefore be construed as exhibiting a high resistance potential. The question of which, if any, antibiotics should be categorised as having high or low potential for selecting resistance has been much debated [5,6,7,8,40]. Generally, narrow spectrum antibiotics are lower risk while broader spectrum agents are higher risk, though there may be exceptions [2,3,5,40]. The term ‘broad spectrum’ can be ambiguous [41,42]. Breadth of spectrum has historically been defined in terms of Gram-stain and clinical versatility, wherein the spectrum of activity incorporates any organisms against which a given agent is active at clinically achievable concentrations [41,42]. It does not automatically follow, however, that the extent to which a given antimicrobial selects problematic resistance or perturbs the microbiome is proportional to its breadth of spectrum as judged by this metric [6,7,40,41,42]. As a case in point, doxycycline has been advocated in recent antimicrobial guidelines as a drug with comparatively limited adverse ecological impact and a minimal risk of promoting *C. difficile* overgrowth [43,44,45,46,47,48,49]. While doxycycline poses less risk than some other agents, it, like other tetracyclines, is assuredly not ‘narrow spectrum’ in the conventional sense as outlined here [49]. Indeed, the term ‘broad spectrum’ was first coined in the 1940s to describe the first tetracyclines and chloramphenicol, given their expansive utility to treat infections due to both Gram-positive and Gram-negative pathogens [49,50,51,52]. Another example of an antibiotic with comparatively low resistance potential despite possessing broad activity is piperacillin-tazobactam. This drug has a wider spectrum of antibacterial action than third generation cephalosporins yet relatively speaking demonstrates a lower propensity for selecting *C. difficile*, VRE and Enterobacterales harbouring extended spectrum β-lactamases (ESBLs) and/or derepressed AmpC β-lactamases [53,54,55,56,57,58,59]. The World Health Organization (WHO) has adapted the concept of resistance potential in the form of the Access, Watch, Reserve (AWaRe) schema which stratifies antimicrobials by risk [1,2,3,4,5]. Access antibiotics are those recommended by the WHO for the routine management of infections [1,2,3,4,5]. They are considered to have a low resistance potential. Watch agents are generally broader spectrum with higher resistance potential and recommended only when access agents are unsuitable, for instance because of allergy or resistance. Reserve agents are used only for multidrug resistant infections, where the use of drugs from the former two categories is precluded [1,2,3,4,5]. Reserve agents are often expensive, newer and often have high toxicity and/or resistance potential [1,2,3,4,5]. Drugs from the same chemical class might have divergent AWaRe classifications [1,2,3,4,5]. The legitimacy of individual AWaRe groupings has been questioned [40]. Most macrolides, for instance, are allocated to the watch group despite having an arguably narrower spectrum of activity than some access drugs, such as amoxicillin-clavulanate [40,60]. Likewise, some argue against the inclusion of lincosamides and nitroimidazoles in the access group as their activity against anaerobes has been linked with microbiome disruption and reduced resilience to colonisation by resistant organisms including VRE and MDR-GNB [40,61,62,63,64,65,66,67,68,69]. Furthermore, lincosamides have the strongest association with *C. difficile* colitis of any antibiotic class deployed clinically [70,71,72]. The European Antimicrobial Resistance Surveillance Network (EARS-NET) and European Surveillance of Antimicrobial Consumption Network (ESAC-NET) curate population level data over time and throughout the European Economic Area (EEA). EARS-NET monitors resistance to antimicrobials amongst invasive isolates of sentinel pathogens, whereas ESAC-NET tracks usage of systemic antimicrobials [73,74]. Together, data from these networks can be interrogated to determine whether spatiotemporal correlations between antimicrobial consumption and resistance exist. Using univariate and multivariate regression analyses, this was assessed methodically for 29 EEA countries.

## 2. Methods

Antimicrobial consumption data in the form of defined daily doses per 1000 inhabitants per day (ddd/1000/day) were collated from ESAC-NET for 29 countries using 18 Anatomical Therapeutic Chemical Classification (ATCC) Codes. ATCC codes corresponded to antibiotic classes as follows: J01 total systemic antibacterials, J01A tetracyclines, J01CA extended spectrum penicillins, J01CE β-lactamase labile penicillins, J01CF β-lactamase stable penicillins, J01CR extended spectrum penicillin/β-lactamase inhibitor combinations, J01DB first generation cephalosporins, J01DC second generation cephalosporins, J01DD third generation cephalosporins, J01 DH carbapenems, J01E sulphonamides and trimethoprim, J01FA macrolides, J01FF lincosamides, J01G aminoglycosides, J01M quinolones, J01XA glycopeptides, J01XD nitroimidazoles and J01XE nitrofurans. Note that for tetracyclines (J01A), sulphonamides and trimethoprim (J01E), and quinolones (J01M), consumption was resolved only to ATCC level 3, while other classes were subdivided down to ATCC level 4. Data were not available at ATCC level 5, corresponding to individual compounds. The 29 nations included were as follows: Austria, Belgium, Bulgaria, Croatia, Cyprus, Denmark, Estonia, Finland, France, Germany, Greece, Hungary, Iceland, Ireland, Italy, Latvia, Lithuania, Luxembourg, Malta, Netherlands, Norway, Poland, Portugal, Romania, Slovakia, Slovenia, Spain, Sweden and the United Kingdom. Mean use as ddd/1000/day/ATCC code was quantified for each of these 29 countries over 4 periods: 2017–2018, 2018–2019, 2019–2020 and 2020–2021. The mean values over two-year periods were used to account for temporal lags in resistance behind fluctuations in consumption. Percentages of invasive isolates in each country over the years 2018, 2019, 2020 and 2021 were recorded for resistotypes relevant to each of five pathogens: *Escherichia coli* (*E. coli*), *Klebsiella pneumoniae* (*K. pneumoniae*), *Streptococcus pneumoniae* (*S. pneumoniae*), MRSA and VRE. Fourteen resistotypes were considered as follows: For *E. coli*: aminopenicillin (AMPR), third generation cephalosporin (3GCR), fluoroquinolone (FQR), aminoglycoside (AGR), and triple resistance to third generation cephalosporin, fluoroquinolone and aminoglycoside (3XR). For *K. pneumoniae*: carbapenem resistance (CARBR), 3GCR, FQR, AGR, 3XR. For *S. aureus*: methicillin resistance (MRSA). For *Enterococcus faecium*: vancomycin resistance (VRE). For *S. pneumoniae*: penicillin nonsusceptibility (PNS-SP) and penicillin nonsusceptibility with erythromycin resistance (PNS/ER-SP). Consumption data for Austria were only available for the 2019–2020 and 2021–2022 periods. Consumption data for Czechia was only available for 2021–2022 and UK consumption data were available for 2017–2018 and 2018–2019 only. Data for AMPR *E. coli* were not available from Sweden. Data for resistance in *S. pneumoniae* were not available for Cyprus, Greece or Malta and only available for one season in Iceland. Univariate and multivariate regressions were modelled in Microsoft Excel spreadsheets for each resistotype in each pathogen versus usage in ddd/1000/day for each country. Backwards stepwise selection was used to select variables which best fit final multivariate regression models for each species and resistotype spanning the whole 5-year time series and 29 nations. Antimicrobial classes represented by each ATCC code were ranked by their correlation *R*, from lowest to highest risk for each resistotype in each pathogen, and are presented in tabulated format for the univariate and multivariate analyses. Sample sizes were calculated a priori to ensure the power of each multivariate model was ≥ 0.8. Variance inflation factors (VIF) were calculated for each multivariate model to rule out multicollinearity. All VIF were ≤5. A subset of seven countries with decreases in ≥2 resistotypes and no increase in MRSA, VRE or any Gram-negative resistotype were considered to have stable or decreasing resistance and were designated as ‘group 1’ countries. Another subset of eight countries were identified as having stably high or rising resistance if they experienced increases in ≥2 resistotypes spread across at least 2 sentinel pathogens and these were designated as ‘group 2’ countries. Median levels of resistance were charted for comparison between group 1 and group 2 countries, as the percentage of isolates displaying each resistotype for the first (2017–2018) and last (2020–2021) seasons studied. Likewise, mean consumption of each antimicrobial class in group 1 and in group 2 countries for the first (2017–2018) and last (2020–2021) seasons was charted, for comparison, in ddd/1000/day.

## 3. Results

### 3.1. Resistance in Group 1 and Group 2 Countries

**Figure 1:**
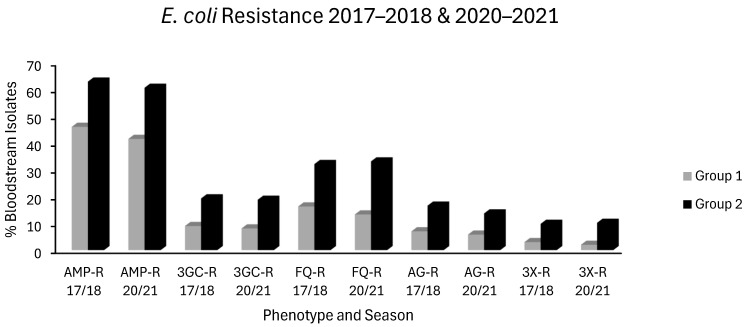
Levels of *E. coli* resistance in group 1 countries (grey) and group 2 countries (black) for the first (2017–2018) and last (2020–2021) seasons of EARS-NET data analysed chronologically.

**Figure 2:**
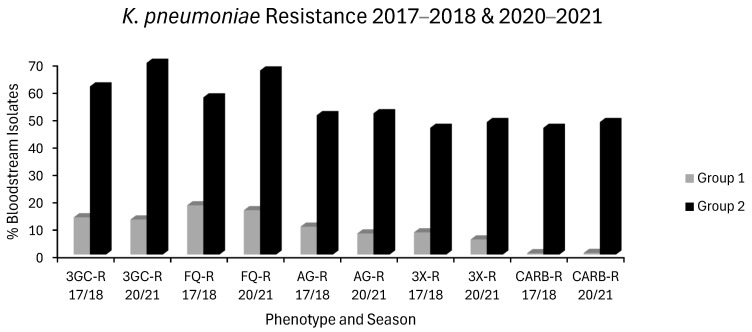
Levels of *K. pneumoniae* resistance in group 1 countries (grey) and group 2 countries (black) for the first (2017–2018) and last (2020–2021) seasons of EARS-NET data analyzed chronologically.

**Figure 3:**
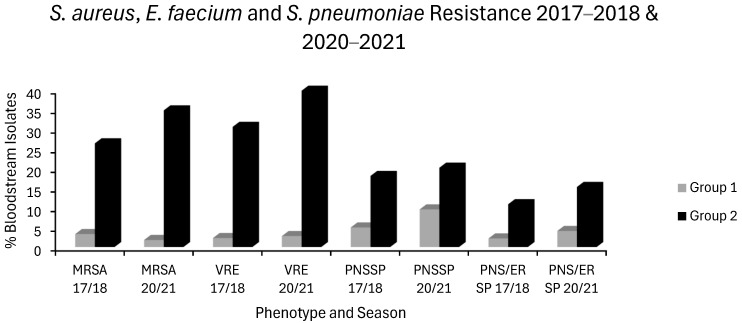
Levels of *S. aureus*, *E. faecium* and *S. pneumoniae* resistance in group 1 countries (grey) and group 2 countries (black) for the first (2017–2018) and last (2020–2021) seasons of EARS-NET data analysed chronologically.

Seven countries experienced no increase in MRSA, VRE or in any of the *E. coli* or *K. pneumoniae* resistotypes and were categorized as group 1 countries. These countries were Belgium, Denmark, Estonia, France, Ireland, Netherlands and Norway. Eight countries had increases in ≥2 resistotypes spread across at least 2 sentinel pathogens and were defined as group 2 countries. These countries were Bulgaria, Croatia, Cyprus, Greece, Hungary, Poland, Romania and Spain. Median resistance levels of each resistotype for the first (2017–2018) and final (2020–2021) seasons studied are charted below for both group 1 and group 2 countries: *E. coli* (Figure 1), *K. pneumoniae* (Figure 2), VRE, MRSA and *S. pneumoniae* (Figure 3). In general, resistance was much higher at baseline in group 2 countries with a tendency to increase overtime. Contrastingly, resistance was much lower at baseline in group 1 countries with a tendency to remain stable or decrease slowly over time.

Other countries, though included in the regression analyses, were not classified as group 1 or group 2 as they did not fit the rigid definitions used to capture dynamic changes in resistance over time. In some cases, this was because of an isolated rise in a single resistotype against a background of generally low and declining resistance. Finland and Sweden, for example, had very low resistance overall, even compared to some group 1 countries, but were not themselves assigned to group 1 due solely to isolated increases in AGR *K. pneumoniae* and MRSA, respectively. Similarly, Germany and Slovenia were excluded from group 1 because of isolated increases in VRE. Italy and Portugal had generally high resistance levels at baseline but achieved significant reductions over the analysed period for most of these resistotypes, other than VRE and CARBR *K. pneumoniae* for which they showed respective increases. Austria, Czechia and the UK were not categorised as full consumption data were not available for these countries. Note that no country achieved a sustained reduction in either of the pneumococcal resistotypes.

### 3.2. Associations Between Overall Antibiotic Use and Resistance

Strong associations were detected between all 14 resistotypes and overall antibiotic consumption in the univariate analysis, with effect sizes ranging from 0.392 for VRE to 0.666 for MRSA (Table 1). In multivariate analyses, this remained significant for 3GCR and 3XR in *E. coli*, for 3GCR, AGR and 3XR in *K. pneumoniae*, and for penicillin nonsusceptibility with or without erythromycin co-resistance in *S. pneumoniae* (Table 2). At the beginning of the analysed period in 2017–2018, overall antibiotic consumption varied from a low of 9.755 ddd/1000/day in the Netherlands to a high of 34.176 ddd/1000/day in Greece. This decreased over time to a low of 8.431 ddd/1000/day in the Netherlands to a high of 26.979 ddd/1000/day in Cyprus for the season of 2020–2021. This >3-fold disparity in antibiotic consumption may imply that there is much scope for countries with high consumption to reduce use. Indeed, Greece, the country with highest consumption at the outset of the analysis, had decreased overall consumption by approximately one-third by the conclusion of the analysis period, from 34.176 ddd/1000/day to 25.829 ddd/1000/day.

### 3.3. Associations Between Tetracycline Use and Resistance

**Figure 4:**
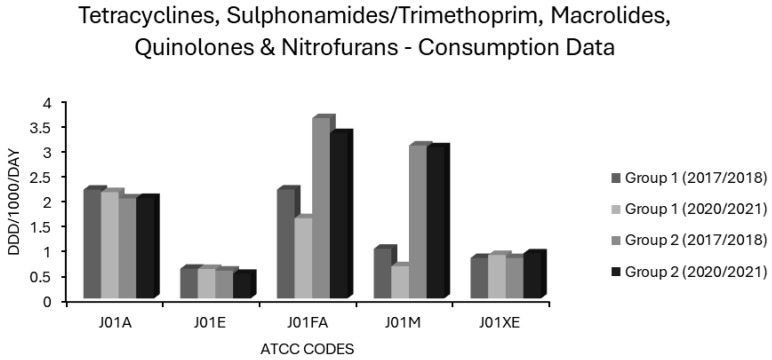
Consumption of tetracyclines, sulphonamides/trimethoprim, macrolides, quinolones and nitrofurans (ATCC codes J01A, J01E, J01FA, J01M and J01XE, respectively) in group 1 and group 2 EEA countries.

Tetracycline use differed little between group 1 and group 2 countries (Figure 4). Usage of tetracyclines had weakly negative correlations in the univariate analysis with all resistotypes other than VRE, PNS-SP, and PNS/ER-SP, for which the associations were weakly positive albeit statistically insignificant (Table 1). The weakly negative correlations found in the univariate analysis for tetracycline use were significant only in the case of 3GCR, FQR and 3XR in *K. pneumoniae* and for MRSA (Table 1). In the multivariate analysis, the weakly negative associations with AMPR in *E. coli* and FQR in *K. pneumoniae* were found to be independently significant and the weakly positive associations with PNS and PNS/ER in *S. pneumoniae* became significant (Table 2). It is not clear why tetracyclines, having a substantial Gram-negative spectrum, appear to be inversely associated with resistance in *E. coli* or *K. pneumoniae*. Determinants of tetracycline resistance have long been known to co-localise with other resistance genes on mobile genetic elements and spread readily, amplified by co-selection from tetracyclines and other agents [75,76,77,78,79]. A possible explanation is that these primarily bacteriostatic agents exert, relatively speaking, a weaker selective pressure than alternative drugs. In other words, the relationship is determined by what tetracyclines are being used instead of rather than by a lack of selective pressure from tetracyclines per se. The low resistance potential of extended spectrum penicillins (J01CA), first generation cephalosporins (J01DB), and sulphonamides/trimethoprim (J01E), all intrinsically active against coliform organisms to some extent, might be similarly explained. Two recent studies of travellers to tropical areas with very high prevalences of ESBL-producing Enterobacterales demonstrated that daily, oral use of 100mg of doxycycline for malaria prophylaxis was not an independent risk factor for gut colonisation by these organisms, whereas use of other antibiotics including fluoroquinolones, macrolides and β-lactams, was. [80,81,82]. Another study recently evaluated the use of doxycycline postexposure prophylaxis (PEP) for bacterial sexually transmitted infections and found no increase in carriage of ESBL-producing coliforms amongst the study population, though tetracycline resistance did increase amongst incident gonorrhoea cases [83,84]. The majority of J01A use in the EEA comprises doxycycline, which may skew these results, and it is possible that other, less used tetracyclines differ from doxycycline in resistance potential [74]. Indeed, it has previously been found that doxycycline selects less readily for resistance amongst commensal *E. coli* than does tetracycline itself, presumably because the latter is less completely absorbed from the gut lumen with consequently greater exposure to the colonic flora [85]. The results obtained here support the inclusion of doxycycline, if not other tetracyclines, in the WHO access category [1,2,3,4,5]. Recent evidence indicates that doxycycline compares favourably to other agents in mild to moderate hospital acquired pneumonia (HAP) and may spare the use of agents with higher resistance potential such as piperacillin/tazobactam, broad spectrum cephalosporins or carbapenems, allowing them to be reserved for moderate to severe HAP where MDR-GNB are more likely to be implicated and the stakes of early clinical failure are unacceptably high [44,86]. At least in areas with low pneumococcal resistance, doxycycline is a useful alternative agent to penicillins for community acquired pneumonia (CAP) and may be preferable to macrolides given its better coverage of *S. pneumoniae* and *Haemophilus influenzae*, lower resistance potential, low risks for cytochrome P450 mediated drug interactions and lack of torsadogenic QTc prolongation [43,45,47,49,87]. In atypical pneumonia, the coverage of *Mycoplasma pneumoniae* and *Chlamydophila* spp. offered by doxycycline is comparable to that provided by macrolides and likely greater where macrolide resistant *M. pneumoniae* is endemic [87,88]. Although active against *Legionella pneumophila*, doxycycline is less potent against this organism than are newer macrolides or fluoroquinolones [89]. Given the high morbidity and mortality associated with Legionnaires’ disease, the latter should probably be prescribed preferentially in atypical pneumonia where this is a suspected or proven aetiology [89,90]. If non-*pneumophila Legionella* spp. is involved, this is even more critical given that some species, notably *L. longbeachae*, are inherently resistant to tetracyclines [91]. As tetracyclines remain active against some MDR-GNB, interest in their use for urinary, respiratory and other infections by these organisms has been rekindled over the last decade. Several reports of clinical success with use of doxycycline and minocycline in such infections have been published [92,93]. There have even been instances where doxycycline was used successfully in urinary tract infections (UTIs) caused by *Pseudomonas aeruginosa*, a pathogen intrinsically resistant to tetracyclines [93]. This highlights the underappreciated fact that susceptibilities are typically reported by laboratories based upon concentrations that are readily attainable in serum [93]. Tetracyclines and many other antibiotics that undergo renal elimination, penicillins included, may reach peak concentrations in urine ≥2 orders of magnitude greater than in serum, overpowering ‘resistant’ pathogens in uncomplicated UTIs [94,95,96]. The consideration of higher urinary breakpoints has the potential to broaden treatment options and improve antimicrobial stewardship [94,95,96].

### 3.4. Associations Between Penicillin Use and Resistance

**Figure 5:**
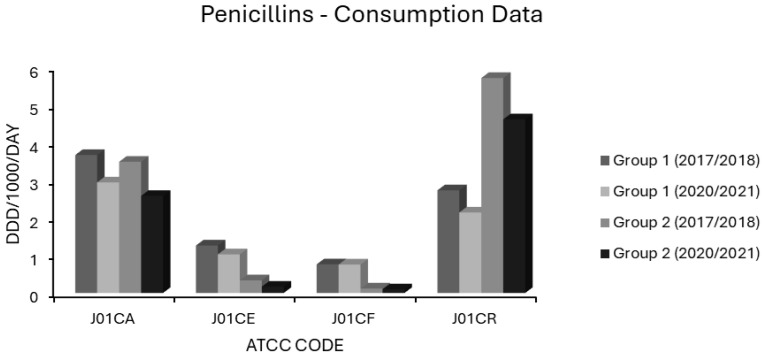
Consumption of penicillins (ATCC code J01C) in group 1 and group 2 EEA countries.

Extended spectrum penicillin (J01CA) use was high in most EEA countries. In group 1 countries, J01CA consumption fell from 3.671 to 2.943 and in group 2 countries from 3.492 to 2.583 ddd/1000/day (Figure 5). Consumption of these drugs, which includes the aminopenicillins ampicillin and amoxicillin, was associated with nonsusceptibility to penicillin and erythromycin in *S. pneumoniae* on univariate analysis (Table 1). Upon multivariate analysis, extended spectrum penicillin use showed weak negative associations with MRSA, VRE and FQR *E. coli*, a moderate positive association with penicillin nonsusceptibility in *S. pneumoniae* and a weak positive association with AMPR *E. coli*, all independently significant (Table 2). The small effect size observed for AMPR *E. coli* might reflect the fact that all of the countries had exceedingly high levels of both aminopenicillin use (Supplementary Table S1) and AMPR in *E. coli* (Figure 1). The finding that aminopenicillin use was not an independent predictor of other resistotypes supports the assignment of these drugs to the WHO access category [1,2,3,4,5]. Nevertheless, aminopenicillin consumption has been documented as a risk factor for emergent resistance not only to aminopenicillins themselves but also to staple drugs for UTI treatment, including trimethoprim, amongst uropathogenic *E. coli* [96,97,98,99]. The resistance potential of aminopenicillins is evidently higher than that of β-lactamase labile penicillins (J01CE), which include benzylpenicillin (penicillin G) and phenoxymethylpenicillin (penicillin V). These narrower spectrum agents, unlike aminopenicillins, were not associated with AMPR in *E. coli*, or any resistotype in univariate or multivariate analyses (Table 1 and Table 2). In group 1 countries, the use of J01CE drugs declined from 1.264 to 1.028 and in group 2 countries from 0.337 to 0.169 ddd/1000/day (Figure 5). Penicillins G or V should be used preferentially where Gram-negative coverage is unnecessary. Amongst the countries with the lowest levels of AMPR *E. coli* were the Nordic nations, which had high utilisation of β-lactamase labile penicillins (Supplementary Table S1). In contrast to other EEA nations, they continue to use penicillins G and V, rather than amoxicillin, as first line therapy for CAP, pharyngotonsillitis, otitis media (OM) and dentoalveolar infections [100,101]. It has been suggested that these drugs exert less selective pressure than aminopenicillins for resistance in Enterobacterales and should be used in preference to them whenever possible [100,101]. Detractors from this position argue that amoxicillin has the advantages of activity against *H. influenzae*, alongside greater bioavailability and palatability when given orally [101,102]. Rhedin and colleagues assessed outcomes in CAP amongst Swedish children aged 1 to 5 years using penicillin V or amoxicillin and found treatment failures were higher with penicillin V (7.7%) versus amoxicillin (4.7%) [102]. Nevertheless, there was no difference in the incidence of serious complications or mortality between the groups, and the number needed to treat with amoxicillin to prevent one clinical failure was large at 31 [102]. The same group demonstrated the noninferiority of oral penicillin V in adult CAP patients with CRB-65 scores ≤ 1 and of intravenous (IV) penicillin G in those with CRB-65 scores of 2 [103]. It is possible that oral penicillin V may be at a disadvantage when compared to oral amoxicillin in countries where penicillin resistant *S. pneumoniae* are more commonly encountered than in Scandinavia [104]. One study attempted to address this hypothesis in Spain, a country with a high prevalence of penicillin resistance, and found penicillin V inferior in intention to treat but not on per protocol analyses [104]. Those investigators, however, cautioned that their study had been underpowered, as they struggled to recruit an adequately large sample cohort [104]. The activity of penicillin G against *H. influenzae*, though weaker than that of aminopenicillins, is greater than that of penicillin V. Thegerström and coworkers found that IV penicillin G did not achieve worse outcomes than IV aminopenicillins in pneumonia cases from which *H. influenzae* was isolated, although they did note a trend towards slower clinical response with penicillin G [105]. It might be argued that subsets of patients with chronic obstructive pulmonary disease (COPD) or bronchiectasis, who are known to be especially susceptible to colonisation and infection with *H. influenzae*, should receive targeted treatment against that organism [106]. A recent study in the UK found, however, that aminopenicillin resistance in *H. influenzae* isolates from COPD patients was already high at 67% [106]. COPD patients are also liable to infection by various other organisms including *Moraxella catarrhalis*, almost invariably resistant to aminopenicillins via β-lactamase production [107]. The Gram-negative spectrum of aminopenicillins has already been much eroded by acquired resistance, but amoxicillin remains an option for the definitive treatment of infections caused by *E. coli*, *Proteus mirabilis*, *Salmonella* and *H. influenzae* strains with laboratory proven sensitivity [108]. It is difficult to envisage a scenario where IV aminopenicillins would be broadly superior to IV penicillin G in empirical therapy. When either is blindly chosen for an undifferentiated infection nowadays, it is with the intention of covering Gram-positive pathogens, a purpose for which aminopenicillins have a needlessly broad spectrum. The use of penicillin G for streptococcal/enterococcal cover would therefore be more appropriate, with an aminoglycoside added, if necessary, for aerobic Gram-negative coverage. Much has been made of the fact that enterococci have lower minimum inhibitory concentrations (MICs) for aminopenicillins than for penicillin G [108,109]. The absolute difference in activity is small, equating to an average of one doubling dilution, and has never been convincingly shown to have any clinical impact, at least for ‘wild type’ strains with MICs below the epidemiologic cut-off [108,109,110,111]. Penicillin G has the advantage of being more stable than aminopenicillins in solution, making it potentially more convenient to administer, particularly in the contexts of using prolonged/continuous infusion to maximise time-dependent bactericidality or in outpatient parenteral antimicrobial therapy (OPAT) [111,112]. Misleadingly, mecillinam (amdinocillin) and its orally administered pivaloyl ester, pivmecillinam, are grouped with the aminopenicillins under ATCC code J01CA, extended spectrum penicillins, as is temocillin [74]. Both mecillinam and temocillin differ substantially from aminopenicillins, not least by their near total lack of activity against Gram-positive organisms and obligate anaerobes [113,114,115,116,117,118]. Each has a spectrum of action limited to Enterobacterales with little cross-resistance to aminopenicillins [113,114,115,116,117,118]. Pivmecillinam has been established as a frontline treatment for uncomplicated bacterial cystitis in Nordic countries since the 1970s with minimal associated resistance, and it accounts for a substantial proportion of J01CA consumption there [119]. Though not an officially approved indication, some data suggest mecillinam may concentrate sufficiently in the renal parenchyma to be effective even in upper UTIs with bacteraemic overspill [120,121]. Elsewhere, J01CA usage is almost entirely comprised of aminopenicillins, hence the resistance potentials of mecillinam and temocillin cannot be inferred from these data [74]. This is unfortunate, since both agents have several ideal characteristics, and warrant investigation for wider applicability in invasive enterobacterial infections. These include bactericidality, low rates of resistance in target pathogens, minimal toxicity, a narrow spectrum conferring lower propensity to distort the gut flora, and in the case of mecillinam, frequent synergy with other β-lactams via complementary binding of different transpeptidases [113,118,119,122,123]. The use of β-lactamase stable penicillins (J01CF), comprising the narrow spectrum antistaphylococcal penicillins (cloxacillin, flucloxacillin and dicloxacillin), was not associated with any resistotype in a univariate analysis (Table 1). Use of β-lactamase stable penicillins (J01CF) had independently negative correlations with MRSA and with 3GCR, AGR, FQR and 3XR in *K. pneumoniae* in a multivariate analysis (Table 2). This may reflect unavailability or prohibitive pricing in some EEA countries including Bulgaria, Hungary, Lithuania and Slovakia (Supplementary Table S1) [74]. As a result, broad spectrum alternatives such as cephalosporins (J01D) and penicillin/β-lactamase inhibitor combinations (J01CR) will likely have been substituted for skin and soft tissue infections (SSTI) with resultant increases in collateral resistance. Lending credence to this argument, use of J01CF agents was 6-to-7-fold lower in group 2 than in group 1 countries (Figure 5). Use of prolonged or continuous infusions given via elastomeric devices and co-administration of probenecid with both oral and IV formulations have been proposed as methods which may improve the pharmacokinetic profile of various narrow spectrum penicillins [111,112,124,125,126]. Such strategies obviate the need to use longer acting but needlessly broad spectrum agents, such as ceftriaxone, in OPAT regimens for staphylococcal and streptococcal infections. The consumption of penicillin/β-lactamase inhibitor (J01CR) agents was found to be high in most EEA countries, and in many of these surpassed the use of narrower spectrum penicillins belonging to the J01CA, J01CE and J01CF groups (Supplementary Table S1). Mean baseline consumption of these drugs was over twice as high in group 2 countries at 5.72 ddd/1000/day than in group 1 countries (2.73 ddd/1000/day) in 2017–2018 (Figure 5). Although both groups had reduced consumption by 2020–2021, this was by <20% in each case (Figure 5). Use of penicillin/β-lactamase inhibitor combinations (J01CR) had positive associations of varying strength with all 14 resistotypes in univariate analysis (Table 1), but independent significance on multivariate analysis held only for MRSA, both resistotypes in *S. pneumoniae*, CARBR in *K. pneumoniae* and FQR and AMPR in *E. coli* (Table 2). Extended spectrum penicillin/β-lactamase inhibitor combinations were more strongly associated with AMPR *E. coli* than were unpotentiated aminopenicillins (Table 1 and Table 2). This may result from the selection of strains hyperproducing penicillinases such as TEM-1 [127,128]. Paradoxically, inhibitor combinations may exert stronger selective pressure than unprotected penicillins, if only strains expressing levels of β-lactamase sufficient to overcome enzymatic inhibition gain a survival advantage [127,128]. Co-resistance to fluoroquinolones may be present in such strains, explaining the strong association observed between FQR in *E. coli* and use of class J01CR agents [129,130]. Carbapenem resistant Enterobacterales have high MICs for extended spectrum penicillin/β-lactamase inhibitor combinations, generally higher than for carbapenems themselves [131]. This could explain the relationship found between CARBR in *K. pneumoniae* and consumption of these drugs (Table 2). While it seems intuitive that extended spectrum penicillin/β-lactamase inhibitor combinations would select for MRSA, the strength of this association was not expected to be so great, given that preferential substitution of these agents for cephalosporins and fluoroquinolones in hospital formularies has been apparently successful in reducing the incidence of nosocomial MRSA infections [131,132,133]. There have been, however, several studies detecting positive associations between extended spectrum penicillin/β-lactamase inhibitor usage and the incidence of colonisation or infection by MRSA [134]. These agents were heavily used throughout the EEA and were the most used systemic antibacterials in many countries, demonstrating that excessive use of any agent can generate resistance (Supplementary Table S1). This presents an obvious though difficult target for stewardship initiatives. There are few clear alternatives for empirical treatment of certain serious infections with diverse bacteriological aetiologies, such as severe HAP. Nevertheless, the wide discordance in use throughout the EEA (Figure 5) would imply that sizeable reductions in usage are theoretically achievable, at least for countries with heavier baseline consumption. Ironically, the increased reliance on extended spectrum penicillin/β-lactamase inhibitors in some countries, including the UK, resulted from earlier admonishments to curtail prescribing of expanded spectrum cephalosporins and fluoroquinolones [131]. While the use of broad spectrum agents such as piperacillin-tazobactam is justified in many HAP cases, evidence dictates that a subset of HAP patients will not require broad Gram-negative cover [44,86]. For intrabdominal sepsis, extended spectrum penicillin/β-lactamase inhibitor combinations will often be appropriate, but a combination of an aminoglycoside and penicillin G (with or without metronidazole based upon the risk of anaerobic involvement) is a reasonable alternative in most cases [135,136,137,138].

### 3.5. Associations Between Cephalosporin Use and Resistance

**Figure 6:**
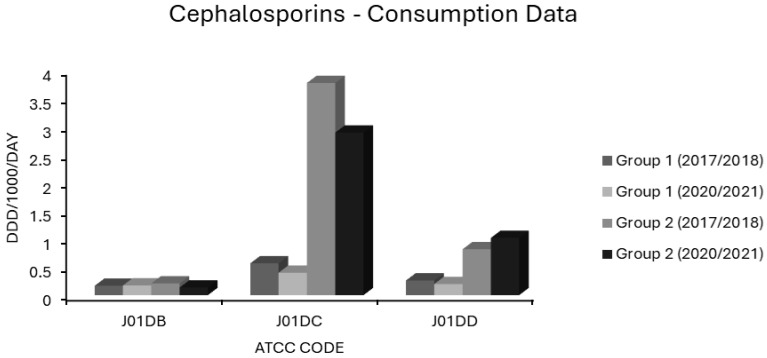
Consumption of cephalosporins (ATCC code J01D) in group 1 and group 2 EEA countries.

In group 1 countries, first generation cephalosporin (J01DB) use was 0.167 and 0.173 ddd/1000/day in 2017/2018 and 2020/2021, and for group 2 countries, 0.21 and 0.135 ddd/1000/day, respectively (Figure 6). Practically all countries had low consumptions of these drugs (Supplementary Table S1). The sole exception was Finland, a country with some of the lowest resistance levels in the EEA (Figure 1, Figure 2, Figure 3 and Figure 4). The use of first generation cephalosporins was not positively associated with any resistotype on univariate analysis (Table 1). In multivariate analysis, J01DB consumption had significant negative correlations with VRE, AMPR *E. coli* and with CARBR, 3GCR and FQR, but not AGR or 3XR, in *K. pneumoniae* (Table 2). This suggests that first generation cephalosporins, unlike second and third generation analogues, have comparatively low resistance potential. This conclusion has also been drawn elsewhere from a recent meta-analysis of data collected at the multinational level [40]. Accordingly, the allocation of first generation cephalosporins to the WHO access group seems valid [1,2,3,4,5]. These drugs merit consideration for the management of infections caused by *E. coli*, *Klebsiella* and *P. mirabilis* strains of established or strongly suspected susceptibility [1,2,3,4,5]. Examples would include the use of agents such as IV cefazolin and oral cefalexin in pyelonephritis or biliary tract infection as definitive treatments or follow-on treatments after the initial use of an IV aminoglycoside while sensitivities are awaited [135,136,137,138,139,140]. Though first generation cephalosporins have less resistance potential than do their second or third generation counterparts, they still have a substantial Gram-negative spectrum, which should not be squandered on uncomplicated SSTIs such as cellulitis and infections of ‘clean’ wounds caused by Gram-positive cocci, where antistaphylococcal penicillins should suffice [135,139,141,142,143]. In patients with genuine penicillin allergies, the cautious use of specific J01DB congeners with R1 side chains lacking cross-reactivity e.g., cefazolin, may be justified in some circumstances [144]. Nitrofurantoin and pivmecillinam are probably better options for treating simple cystitis given that these drugs, unlike first generation cephalosporins, are not generally deemed to be useful in the management of systemic infections [96,97,118,119]. Consumption of second generation cephalosporins (J01DC) was high in many countries but declined over the study period in both groups 1 and 2, from 0.566 to 0.399 and from 3.77 to 2.888 ddd/1000/day, respectively (Figure 6). Mean third generation cephalosporin (J01DD) usage in group 2 countries increased from 0.816 to 1.018 ddd/1000/day but fell from 0.261 to 0.198 ddd/1000/day over the same time for group 1 (Figure 6). Seven of eight group 2 countries had increasing J01DD use, consumption in the remaining country, Spain, was stable (Supplementary Table S1). Both second and third generation cephalosporins were strongly associated with all 14 resistotypes on univariate analysis (Table 1). For second generation agents, these strong associations remained independently significant on multivariate analysis for MRSA, VRE and FQR in *E. coli* and all resistotypes for both pneumococci and *K. pneumoniae* (Table 2). In the case of third generation drugs, significance on multivariate analysis was maintained for all resistotypes other than AMPR in *E. coli*, both *S. pneumoniae* resistotypes and, unexpectedly, VRE (Table 2). Use of second and third generation cephalosporins poses a clear target for antimicrobial policymakers as these agents are being used in clinical situations where narrower spectrum agents with less resistance potential would be more appropriate. Wide disparity in use between countries implies that this should not be an unrealistic undertaking. Based on the analysis presented here, it cannot be determined whether individual second and third generation cephalosporins differ in terms of resistance potential and it is possible that this is the case even though these classes have an overall high risk for resistance selection [26,27,28,29,30,132,133]. Some studies have found that cefotaxime, which undergoes much less biliary excretion than ceftriaxone, exerts less selective pressure upon the bowel flora for *C. difficile* and MDR-GNB though other studies have yielded contradictory findings [145,146,147,148].

### 3.6. Associations Between Carbapenem Use and Resistance

**Figure 7:**
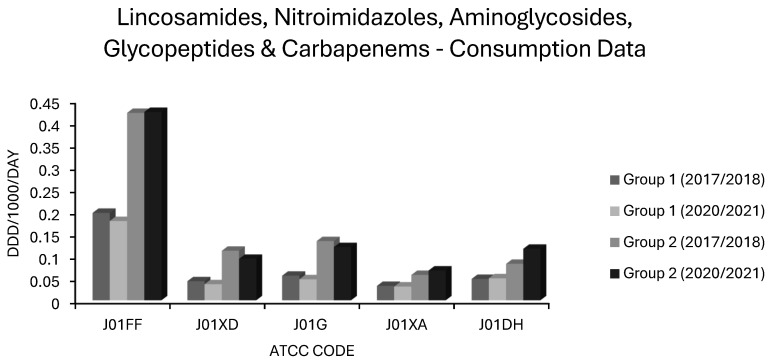
Consumption of lincosamides, nitroimidazoles, aminoglycosides, glycopeptides and carbapenems (ATCC codes J01FF, J01XD, J01G, J01XA and J01DH, respectively) in group 1 and group 2 EEA countries.

Carbapenem (J01DH) use was stable in group 1 countries at ~0.04 ddd/1000/day over 5 years but increased by almost one-third in group 2 countries in the same period from a baseline of 0.0819 ddd/1000/day, already more than double that of group 1 countries (Figure 7). Not surprisingly, carbapenem use correlated strongly with CARBR, 3GCR and FQR in *K. pneumoniae* on multivariate analysis (Table 2). It seems probable that the relationship between carbapenem consumption and CARBR is causal, and conversely, that increased 3GCR and FQR will have fuelled reliance on carbapenems, as has been reported previously [149]. Worryingly, resistance to this crucial class of ‘last resort’ antimicrobials was high and rising amongst *K. pneumoniae* isolates in many countries (Figure 2). By 2021, almost 75% of *K. pneumoniae* isolates from Greece and over half from Romania were carbapenem resistant [73].

### 3.7. Associations Between Sulphonamide/Trimethoprim Use and Resistance

Consumption of sulphonamides/trimethoprim (J01E) differed little between group 1 and 2 countries, remaining stable in both over 5 years (Figure 4). All associations of either polarity were weak and insignificant on univariate analysis (Table 1). Very weak, yet significant, positive associations with VRE and with 3GCR in *E. coli* and a negative relationship of similar magnitude with combined resistance (3XR) in *E. coli*, however, became apparent on multivariate analysis (Table 2). Significant albeit weak negative associations were also evident between J01E use and MRSA as well as for 3GCR, FQR and CARBR resistotypes in *K. pneumoniae* (Table 2). Overall, this suggests that these agents have a low resistance potential and that their inclusion in the WHO access group is warranted [1,2,3,4,5]. Where susceptibility is proven, co-trimoxazole is a valuable option in management of Gram-negative infections of the respiratory, genitourinary, and biliary tracts, or of the abdominopelvic cavity [150]. Considering the wide applicability of sulphonamides/trimethoprim in systemic infections against an increasing background of resistance, blind use for treatment or prophylaxis of UTI no longer appears tenable and would be expected to contribute to further increases in resistance to these valuable agents [96,97,98,99].

### 3.8. Associations Between Macrolide Use and Resistance

Macrolide use was common in most countries (Supplementary Table S1). In group 1 countries, mean J01FA consumption decreased from 2.18 to 1.607 ddd/1000/day and in group 2 countries from 3.609 to 3.31 ddd/1000/day (Figure 4). The consumption of macrolides (J01FA) was strongly associated with all 14 resistotypes on univariate analysis (Table 1), and this retained significance on multivariate analysis for all *S. pneumoniae* and *E. coli* resistotypes and for MRSA, though not for any of the *K. pneumoniae* resistotypes nor for VRE (Table 2). These findings suggest that the allocation of macrolides to the WHO watch category is apt [1,2,3,4,5]. Macrolide use has previously been implicated as a risk factor for colonisation and infection with MRSA, and the detection of an association between macrolide consumption and MRSA incidence is not unexpected [151,152,153,154]. The relationship observed here between macrolide usage and resistance in *E. coli* is more surprising. Enterobacterales have an intrinsically high degree of resistance to macrolides, thus it is conceivable that they would be subject to minimal selective pressure from these agents [155]. The frequent detection of macrolide specific resistance determinants such as *mph* and *erm* genes in Enterobacterales, often linked with other resistance genes on plasmids, implies that this is not the case [155,156]. Some studies have found that macrolide exposure is an independent risk factor for colonisation or infection with MDR-GNB [157,158]. Others report that macrolides have one of the lowest risks amongst antimicrobial classes for resistance selection in Gram-negative pathogens, and some investigators have questioned their place in the WHO watch category based upon this [40,97]. It is unclear why the correlation between macrolide consumption and various resistotypes was independently significant for *E. coli*, *S. pneumoniae* and MRSA but not for *K. pneumoniae* and VRE. Whatever the true resistance potential of macrolides, as an antimicrobial class with limited, well defined first line indications, they are undoubtedly overused. Their main applications are in penicillin allergic patients with CAP or SSTI. It is well established that a true penicillin allergy, albeit potentially life threatening, is massively over diagnosed [144]. Even in CAP patients without a documented allergy, macrolides are oftentimes advocated for use alongside a β-lactam with the rationale that they will cover atypical organisms and may reduce mortality via immunomodulatory mechanisms and/or suppression of bacterial virulence factors such as pneumolysin, a pore-forming exotoxin secreted by pneumococci [159,160,161,162]. It has not been established whether the benefits of adding a macrolide to a β-lactam apply generally to CAP patients or only to subsets with the most severe disease or for whom an atypical aetiology is likely; reports in the published literature are conflicting [163,164]. In many locales, macrolide resistance is now common amongst the principal pathogens causing both CAP (*S. pneumoniae* and *M. pneumoniae*) and SSTI (*S. aureus* and β-haemolytic streptococci) [88,165,166].

### 3.9. Associations Between Lincosamide Use and Resistance

Mean lincosamide (J01FF) consumption remained stable in both group 1 and 2 countries, but was more than twice as high in the latter (Figure 7). Usage levels varied across EEA countries (Supplementary Table S1). Like macrolides, lincosamides were independently associated with all resistotypes in *E. coli,* though only with CARBR in *K. pneumoniae* (Table 2). Lincosamides, unlike macrolides, were not associated with MRSA or with resistance in *S. pneumoniae*. In fact, weak though significant negative correlations were observed for these pairings and with VRE (Table 2). This might relate to the incomplete cross-resistance patterns of macrolides and lincosamides seen in *Staphylococcus* and *Streptococcus* spp. [167]. Resistance to erythromycin, the prototypical 14-membered macrolide, typically confers constitutive cross-resistance to other 14-membered macrolides, and to its 15-membered azo derivative, azithromycin, in these genera. Conversely, cross-resistance to lincosamides and to 16-membered macrolides such as josamycin and spiramycin, is often inducible rather than constitutive, if present at all [167]. The principal lincosamide in clinical use, clindamycin, is counted as an access agent in the WHO AWaRe schema [1,2,3,4,5]. Its independent association with *E. coli* resistance as observed here, along with a substantial risk of provoking *C. difficile* colitis, may imply that this allocation is not deserved. The legitimacy of clindamycin as an access agent has been previously questioned in the literature [40]. Note, however, that although the correlations identified between lincosamide use and *E. coli* resistance did attain independent significance, the effect size was comparatively modest (Table 2).

### 3.10. Associations Between Aminoglycoside Use and Resistance

Average aminoglycoside (J01G) utilisation decreased from 0.064 to 0.052 and from 0.133 to 0.12 ddd/1000/day in the group 1 and 2 countries, respectively (Figure 7). Countries differed widely in aminoglycoside consumption (Supplementary Table S1). Although aminoglycoside (J01G) consumption correlated positively with all resistotypes in the univariate analysis (Table 1), the significance of this was lost in the multivariate analysis, for all but three resistotypes, namely AGR and 3XR in *K. pneumoniae* and AGR alone in *E. coli* (Table 2). It would therefore seem that aminoglycosides, though selecting for resistance to themselves, do not appreciably select for resistance to second/third generation cephalosporins or fluoroquinolones in these pathogens [168,169,170,171]. Conversely, fluoroquinolones and second/third generation cephalosporins appear to select for resistance to aminoglycosides as well as to themselves and to each other [168,169,170,171]. Cross-resistance between individual aminoglycosides, depending on the mechanism involved, is often absent in aerobic GNB. The same cannot be said for quinolones or cephalosporins [168,169,170,171]. Isolates with resistance to gentamicin, for instance, often remain fully sensitive to amikacin depending on the underlying mechanism [171]. EARS-NET classifies isolates as aminoglycoside resistant if they exhibit resistance to any one of gentamicin, tobramycin, netilmicin or amikacin [73]. Prior to the advent of ultra-broad-spectrum β-lactams and fluoroquinolones in the 1980s, aminoglycosides had been preferred ‘workhorse’ agents for severe infections due to aerobic GNB, but they fell out of favour owing to toxicity concerns and the tedious, costly requirement for therapeutic drug monitoring [172,173]. The findings presented here indicate that aminoglycosides have a comparatively low resistance potential and suggest that the assignment of gentamicin and amikacin to the WHO access group is justified [1,2,3,4,5]. In much of the UK and particularly in Scotland, gentamicin has been used first line, with or without amoxicillin, for the empirical treatment of undifferentiated sepsis for over a decade, without compelling evidence of increasing resistance or clinical inferiority [137,138]. The empirical use of an optimally dosed aminoglycoside to provide aerobic Gram-negative coverage for up to 96 h while sensitivities are awaited may spare the need for empirical use of broad spectrum agents in urinary, biliary and intraabdominal sepsis [137,138]. Where ongoing Gram-negative coverage is required beyond this time and the use of further doses is thought to risk toxicity, a targeted agent with low resistance potential can be preferentially chosen based on laboratory results. The use of aminoglycosides was twice as high in group 2 countries as in group 1 countries and changed little over five years in either group (Figure 7). This might relate to their use as alternatives to carbapenems or polymyxins for infections due to MDR-GNB, though this is purely speculative. Some carbapenem resistant GNB remain sensitive to one or more aminoglycosides. For instance, KPC-producing *K. pneumoniae* belonging to the prominent ST-258 clone often retain gentamicin susceptibility [174].

### 3.11. Associations Between Quinolone Use and Resistance

In group 1 countries, mean quinolone (J01M) use was 3-fold lower at baseline relative to group 2 countries (0.994 vs. 3.06 ddd/1000/day) and further declined by approximately one-third over 5 years (Figure 4). Two of the eight group 2 countries, Bulgaria and Cyprus, increased J01M use by 0.824 and 0.928 ddd/1000/day, respectively, from already high baselines of 2.996 and 5.751 ddd/1000/day (Supplementary Table S1), with the overall effect that mean quinolone consumption in the group 2 countries was unchanged over 5 years (Figure 4). In the univariate analysis, quinolones (J01M) were strongly associated with all resistotypes (Table 1) and these associations retained independent significance in multivariate analysis for all resistotypes except 3GCR in *K. pneumoniae*, VRE, both *S. pneumoniae* resistotypes and unexpectedly, MRSA (Table 2). These findings further validate the view that quinolones are major drivers of resistance, which warrants their allocation to the WHO watch group [1,2,3,4,5]. Although they have a very high resistance potential, their advantages of bactericidality, Gram-negative coverage and uniquely high oral bioavailability may tempt clinicians to overuse this class of drugs [175]. The remarkably high resistance potential of quinolones as a class must be weighed against the advantages of oral therapy, which may include avoidance of the need for hospitalisation and vascular access, which are both risk factors in and of themselves for the acquisition of drug-resistant nosocomial infections [176]. While it seems from these findings that quinolones as a class have a high resistance potential and do merit inclusion in the WHO watch category, these data do not permit comparisons of resistance potential between individual quinolones. In vitro studies would suggest that the genetic barrier towards de novo mutational resistance is lower for ciprofloxacin than for moxifloxacin in Gram-positive organisms, whereas the converse holds true for Gram-negative organisms including Enterobacterales and *P. aeruginosa* [177,178]. Some have argued that levofloxacin has a more balanced spectrum and superior pharmacokinetic profile in this regard, with an overall lower resistance potential and lower risk for *C. difficile* colitis, though clear evidence for this in clinical practice is lacking [6,7,70].

### 3.12. Associations Between Glycopeptide Use and Resistance

Mean glycopeptide (J01XA) use increased from 0.057 to 0.067 ddd/1000/day in group 2 countries and stabilised around 0.03 ddd/1000/day in group 1 countries (Figure 7). Consumption of glycopeptides (J01XA) had positive correlations of varying strength and significance for all resistotypes on univariate analysis. However, none of these remained significant in multivariate analysis apart from the association observed with PNS-SP (Table 2). It seems probable that this association results from greater reliance on vancomycin in invasive pneumococcal disease where rates of nonsusceptibility to penicillin and other β-lactams are high. Surprisingly, the association between VRE and glycopeptide consumption was not independently significant (Table 2). The literature on whether glycopeptide exposure is a risk factor for acquisition of VRE or MDR-GNB is conflicting [40,66,134,179,180,181,182,183]. A limitation of this study is that the data were not stratified by route of glycopeptide administration. It is conceivable that orally administered glycopeptides may exert greater selective pressure on the gut flora than their IV counterparts, given that the latter do not appreciably concentrate in the bowel lumen [184,185]. Indeed, it is for this reason that vancomycin administered orally but not intravenously is effective in colitis due to *C. difficile* or staphylococci [185,186]. Conversely, it may also stand to reason that IV glycopeptides would exert greater selective pressure on organisms in other anatomic compartments.

### 3.13. Associations Between Nitroimidazole Use and Resistance

Group 1 countries had a slight drop in nitroimidazole (J01XD) use (from 0.048 to 0.041 ddd/1000/day), as did group 2 countries (from 0.111 to 0.093 ddd/1000/day) (Figure 7). Nitroimidazole (J01XD) use was associated with 12 of 14 resistotypes on univariate analysis, but this retained significance only for VRE and for all 5 *E. coli* resistotypes in multivariate analysis (Table 1 and Table 2). The lack of association between nitroimidazole consumption and MRSA or pneumococcal resistance in multivariate analysis may be due to the fact that *S. aureus* and *S. pneumoniae*, unlike *E. coli* or *Enterococcus* spp., do not reside primarily in the gut alongside a predominantly anaerobic microflora [69,187,188]. The association of lincosamides, another class of antianaerobic agent, with *E. coli* resistotypes but not MRSA or resistant *S. pneumoniae*, might be similarly explained (Table 2). Note, however, that lincosamides were not associated with VRE, unlike nitroimidazoles. Nitroimidazoles were more strongly associated with VRE than any other agents, including glycopeptides and cephalosporins (Table 2). Intriguingly, Spain and Portugal both reported zero usage of nitroimidazoles and very low rates of VRE (Figure 3), despite having both high consumpt ion of other antimicrobial classes (Supplementary Table S1) and a high prevalence of other resistant organisms (Figure 1, Figure 2, Figure 3 and Figure 4). Many studies have previously identified metronidazole use as a risk factor for colonisation with VRE and MDR-GNB [61,62,63,64,65,66,67,68,69]. Strong, independent associations between nitroimidazole use and resistance might suggest that metronidazole is misplaced in the WHO access group [1,2,3,4,5,40]. It should be noted, however, that all of the main antianaerobic agents, including nitroimidazoles, penicillin/β-lactamase inhibitor combinations and lincosamides were found to entail relatively high resistance risk (Table 1 and Table 2). Why lincosamides and nitroimidazoles were associated with resistance in *E. coli* but not in *K. pneumoniae* is unclear given that both are enteric organisms (Table 2). This may be explained by the fact that while both organisms reside predominantly in the gut, *K. pneumoniae* does so less exclusively, and infections due to this organism may result comparatively more often from environmental sources as opposed to autoinoculation or translocation from the host’s own gut [189,190,191,192,193]. *K. pneumoniae* also occurs proportionately more often in nosocomial infections relative to *E. coli,* and it is possible that shortcomings in infection control procedures contribute more to its spread with less influence from antibiotic consumption [191,192,193]. Furthermore, these distinct species may occupy subtly different niches when colonising a host and differ in their interactions, competitive and cooperative, with commensal anaerobes of the alimentary canal [194,195,196]. Given that nitroimidazoles are generally used in conjunction with other agents to provide aerobic coverage, this cannot be excluded as a confounding factor. Hoffman and colleagues found that in patients undergoing colorectal surgery, prophylaxis with a combination of cefuroxime and metronidazole promoted intestinal carriage of Enterobacterales resistant to carbapenems and/or third generation cephalosporins more so than did monoprophylaxis with ertapenem [197]. Regardless, antianaerobic agents are widely overused and should be an easy target for antimicrobial stewardship initiatives [198]. Most oropharyngeal anaerobes are adequately covered by penicillin G or V alone, and routine addition of metronidazole to therapy in peritonsillar abscess or dentoalveolar infections confers no additional benefit providing adequate drainage is achieved [199,200,201]. In aspiration pneumonia, the addition of metronidazole to a penicillin does not lead to better outcomes, yet it is still widespread practice [202,203]. Except in cases complicated by anaerobic bacteraemia or biloenteric anastomoses, anaerobic coverage is not required in biliary tract infections [204]. Crucially, the use of metronidazole is redundant where penicillin/β-lactamase inhibitor combinations, carbapenems, chloramphenicol or tigecycline are used, as these agents all offer broad anaerobic coverage [204,205,206,207,208,209]. Aside from the use of adjunctive clindamycin in necrotising SSTI to suppress exotoxin production by β-haemolytic streptococci, *S. aureus* and histotoxic clostridia, double anaerobic coverage is almost never clinically indicated and has repeatedly been linked to increased harms without added benefit [205,206,210,211,212,213,214,215].

### 3.14. Associations Between Nitrofuran Use and Resistance

Average nitrofuran consumption was similar in group 1 and 2 countries and increased slightly in both over five years (Figure 4). Individual nations ranged greatly in levels of nitrofuran usage with no use recorded at all for Bulgaria or Slovakia and >4 ddd/1000/day recorded at the other extreme in Poland (Supplementary Table S1). The consumption of nitrofurans (J01XE) had no significant associations, positive or negative, with any resistotype, in the univariate analysis (Table 1). Some associations between nitrofuran use and certain resistotypes, although weak, gained significance in the multivariate analysis (Table 2). Specifically, nitrofuran consumption had weak negative correlations with FQR for *E. coli* and CARBR for *K. pneumoniae* while having weak positive correlations with AMPR in *E. coli*, with all *K. pneumoniae* resistotypes other than CARBR and with VRE (Table 2). Nitrofurantoin, though usually effective against *E. coli*, has much less consistent activity against other GNB which may cause urinary tract infections (UTIs), especially among patients who are catheterised or have structural abnormalities of the genitourinary system. Susceptibility of *Klebsiella* and *Enterobacter* is variable whilst *P. aeruginosa*, *Proteeae* and *Serratia* are intrinsically resistant [216,217]. It seems logical that nitrofurantoin use may shift the aetiology of UTI in favour of these organisms, particularly in the case of hospitalised patients with urological risk factors. Increased reliance on nitrofurantoin as one of exceedingly few options for the treatment of UTIs due to VRE may account for the positive relationship observed between this resistotype and nitrofuran consumption [218,219]. It could be argued that since nitrofurans are indicated solely for uncomplicated lower UTIs and are not useful in serious systemic infections there is less at stake from resistance towards them than to other agents and that they should thus be used preferentially for this niche application. Nitrofurantoin use has previously been shown to be inversely correlated with resistance among *E. coli* to other antibiotics commonly used for UTIs, such as trimethoprim [97]. Nitrofurantoin does not adversely impact the gut flora, presumably because it concentrates exclusively in urine [219,220]. On balance, the allocation of nitrofurantoin to the WHO access group seems appropriate [1,2,3,4,5]. The preferential use of nitrofurantoin for treatment of uncomplicated lower UTIs is justified, though complex lower UTIs with risk factors for the involvement of GNB other than *E. coli* may require alternative therapy and this should be guided by susceptibility testing whenever possible.

## 4. Conclusions

Analysis of ESAC-NET and EARS-NET data indicates that there are strong associations between overall antimicrobial consumption and the prevalence of key resistance phenotypes in sentinel pathogens, varying in spatiotemporal distribution. In summary, the consumption of certain agents, namely second and third generation cephalosporins, fluoroquinolones, extended spectrum penicillin/β-lactamase inhibitor combinations, carbapenems, macrolides and nitroimidazoles is strongly associated with antimicrobial resistance in EEA countries. Given that much of this use will be for UTIs, CAPs, and uncomplicated SSTIs, in all of which narrow spectrum agents with lower resistance potential would be better suited, this highlights a clear target for antimicrobial stewardship initiatives. Low resistance potential agents including both β-lactamase labile and stable penicillins (represented by ATCC codes J01CE and J01CF), first generation cephalosporins (ATCC J01DB) and nitrofurans (ATCC J01XE) have been used little, if at all, in certain group 2 countries with very high resistance levels, possibly due to unavailability or prohibitive costs. Addressing inequitable access to such agents may contribute to improvements in antimicrobial stewardship with consequent reductions in antimicrobial resistance. Aminopenicillins, though having a low resistance potential overall, select for resistance towards amoxicillin/ampicillin, and likely also to other agents such as trimethoprim, in *E. coli*. Though this may not seem a significant problem given that resistance to these agents has now been widespread for decades, it should be noted that the use of these antibiotics is extremely common, as are *E. coli* infections of the urinary and biliary tracts, abdominal cavity and bloodstream. A substantive decrease in use of aminopenicillins may therefore yield a significant decline in selective pressure for resistance in *E. coli* and presumably also in other pathogens innately sensitive to these drugs such as *P. mirabilis*, *Helicobacter pylori* and *H. influenzae*. Where aminopenicillins are not being utilised specifically for their Gram-negative activity, preferential substitution of penicillin G or V, as is the practice in Scandinavian countries, merits consideration. All agents with broad antianaerobic activity have a high resistance potential, with the implication that clinicians and antimicrobial policymakers should carefully consider where anaerobic coverage is needed to avoid unnecessary use of such agents singly or worse still, in redundant combinations. A key strength of this study is its use of a large sample size extracted from data that are freely available in the public domain, with power calculated a priori. Though countries outside the EEA were not considered here, it is probable that many of the findings presented could be extrapolated more widely. While one would not wish to stifle an already lacklustre antimicrobial pipeline, it can be noted that many agents in clinical development at present belong to already known antimicrobial classes with overall high resistance potential such as the quinolones, cephalosporins and carbapenems [221]. This could indicate a repeating historical precedent whereby wider spectrum agents are favoured over narrower spectrum agents. During the last major flurry of antimicrobial development in the 1970s–1980s, for example, fluoroquinolones and third generation cephalosporins were readily embraced by medics while narrower spectrum agents such as temocillin and mecillinam, effective solely against Enterobacterales, and cefsulodin, exclusively targeting *P. aeruginosa*, were commercial failures [113,114,118,222,223]. It would seem prudent going forward to closely monitor new agents for resistance as they are introduced and to evaluate their microbiotoxicity [224]. Alternative approaches including bacteriophage therapy, bacteriocins, antivirulence compounds, antibodies and immunomodulators have all shown promise in treating infection with a potentially smaller ecological footprint than conventional small molecule antibiotics, though rapid and accurate diagnostics may be necessary to facilitate the clinical application of many of these agents given the high selectivity of their actions [225,226,227,228]. A key limitation of the study is the inability to definitively determine whether the correlations detected were truly causal, though temporal changes in resistance following changes in consumption trends suggest that they may be. In some cases, associations between resistotype and consumption of a drug class may signify effect rather than cause. A further weakness of this study is that the consumption of individual compounds was not considered (ATCC level 5). Thus, differences in resistance potential between various agents belonging to the same class could not be resolved. Factors such as population density, pneumococcal vaccination, travel/migration, infection control measures, use of antimicrobials in veterinary medicine/agriculture and the COVID-19 pandemic were not included in these models, and the possibility that they have confounded the results to some extent cannot be discounted. A further limitation is that certain less-used antimicrobial classes were not considered at all. Examples include the polymyxins, amphenicols, streptogramins, oxazolidinones, monobactams, phosphonics, rifamycins and fusidanes [74]. It is also possible that the resistance potential of an agent could change over time in accordance with evolving microbial aetiologies and resistance patterns. As an illustration of this, fluoroquinolone use is now thought to pose a substantial risk for acquisition of *C. difficile,* with almost universal consensus [38,229]. This was not always the case, and it is now believed that the acquisition of fluoroquinolone resistance by key ribotypes with enhanced virulence, such as 027, changed the epidemiologic situation from one where quinolones were associated with a comparatively minimal hazard to the higher risk that is widely acknowledged today [38,229]. Similarly, tetracyclines are now generally thought to carry a minimal risk of selecting MRSA. This was not always so, and it owes to the fact that currently circulating strains happen to be tetracycline sensitive. The predominant MRSA clones causing problems in the 1960s and 1970s were resistant to tetracycline, and rampant overuse of these drugs at that time was thought to have contributed to their success [230]. These limitations present opportunities for future studies delineating risk factors for the spread of multidrug resistant organisms.

## Figures and Tables

**Table 1:** Associations between 14 resistotypes and antimicrobial classes as determined in univariate regression. Statistically significant (*p* < 0.05) associations are marked with an asterisk *. Associations are mapped as Pearson’s correlation *R* in order of lowest risk to highest risk.

3GCR EC	*R*	FQR EC	*R*	AGR EC	*R*	3XR EC	*R*	AMPR EC	*R*	VRE	*R*	**PNS** **SP**	***R***
J01CF *	−0.487	J01CF *	−0.502	J01CF *	−0.403	J01C *	−0.483	J01CE *	−0.442	J01CE *	−0.273	J01CE *	−0.23
J01CE *	−0.429	J01CE *	−0.484	J01CE *	−0.382	J01C *	−0.402	J01DB *	−0.4	J01CF *	−0.252	J01CF	−0.155
J01DB *	−0.202	J01DB *	−0.276	J01DB *	−0.22	J01DB *	−0.203	J01CF	−0.183	J01DB	−0.12	J01FF	−0.106
J01CA	−0.178	J01CA *	−0.222	J01CA	−0.133	J01CA *	−0.192	J01E	−0.095	J01CA	−0.111	J01XE	−0.046
J01XE	−0.165	J01E	−0.183	J01XE	−0.127	J01A	−0.115	J01A	−0.028	J01FF	−0.014	J01DB	0.04
J01A	−0.114	J01A	−0.144	J01A	−0.086	J01XE	−0.073	J01CA	0.068	J01E	0.057	J01A	0.047
J01E	0.017	J01XE	−0.025	J01E	0.009	J01E	−0.014	J01XE	0.094	J01A	0.115	J01XD	0.057
J01XA *	0.209	J01FF *	0.304	J01XA *	0.23	J01XA *	0.224	J01FF	0.133	J01XE	0.147	J01E	0.058
J01DH *	0.342	J01XA *	0.444	J01DH *	0.335	J01DH *	0.284	J01DH *	0.319	J01DD *	0.238	J01DH *	0.241
J01FF *	0.347	J01G *	0.467	J01CR *	0.361	J01CR *	0.339	J01XD *	0.386	J01G *	0.262	J01G *	0.266
J01CR *	0.39	J01DH *	0.495	J01 *	0.459	J01 *	0.417	J01XA *	0.434	J01CR *	0.293	J01DC *	0.296
J01 *	0.449	J01 *	0.568	J01FF *	0.463	J01FF *	0.46	J01DC *	0.461	J01DH *	0.353	J01FA *	0.304
J01DC *	0.582	J01XD *	0.59	J01XD *	0.527	J01FA *	0.601	J01G *	0.473	J01 *	0.392	J01DD *	0.313
J01FA *	0.601	J01CR *	0.62	J01DC *	0.59	J01G *	0.605	J01DD *	0.532	J01M *	0.442	J01XA *	0.337
J01XD *	0.59	J01DC *	0.652	J01FA *	0.6	J01DC *	0.606	J01 *	0.615	J01XA *	0.485	J01CA *	0.38
J01G *	0.626	J01DD *	0.666	J01DD *	0.654	J01XD *	0.611	J01CR *	0.63	J01FA *	0.494	J01M *	0.39
J01M *	0.73	J01FA *	0.678	J01M *	0.657	J01M *	0.62	J01M *	0.632	J01DC *	0.523	J01CR *	0.522
J01DD *	0.759	J01M *	0.81	J01G *	0.689	J01DD *	0.655	J01FA *	0.697	J01XD *	0.596	J01 *	0.572
**3GCR KP**	***R***	**FQR KP**	***R***	**AGR KP**	***R***	**3XR KP**	***R***	**CARBR KP**	***R***	**MRSA**	***R***	**PNS/ER SP**	***R***
J01CF *	−0.57	J01CF *	−0.581	J01CF *	−0.541	J01CF *	−0.548	J01CF *	−0.299	J01CE *	−0.423	J01CE *	−0.206
J01CE *	−0.51	J01CE *	−0.492	J01CE *	−0.448	J01CE *	−0.443	J01CE *	−0.283	J01CF *	−0.386	J01CF	−0.185
J01DB *	−0.255	J01DB *	−0.268	J01DB *	−0.224	J01DB *	−0.23	J01DB *	−0.193	J01DB *	−0.226	J01FF	−0.046
J01A *	−0.217	J01A *	−0.263	J01A	−0.181	J01A *	−0.189	J01XE	−0.128	J01A *	−0.197	J01XE	−0.012
J01E	−0.071	J01E	−0.099	J01E	−0.067	J01E	−0.083	J01A	−0.04	J01XE	−0.164	J01E	0.022
J01CA	−0.04	J01CA	−0.057	J01CA	−0.019	J01CA	−0.018	J01E	−0.028	J01E	−0.138	J01DB	0.073
J01XE	0.068	J01XE	0.106	J01XE	0.061	J01XE	0.077	J01CA	0.039	J01CA	−0.075	J01A	0.081
J01XA *	0.321	J01XA *	0.341	J01XA *	0.275	J01XA *	0.284	J01FF	0.153	J01FF	−0.016	J01XD	0.091
J01FF *	0.359	J01FF *	0.358	J01DH *	0.394	J01DH *	0.41	J01XD *	0.378	J01G *	0.434	J01DH *	0.223
J01DH *	0.431	J01DH *	0.434	J01CR *	0.408	J01FF *	0.429	J01CR *	0.485	J01XD *	0.442	J01XA *	0.236
J01CR *	0.502	J01CR *	0.504	J01FF *	0.444	J01CR *	0.411	J01DD *	0.504	J01XA *	0.534	J01G *	0.242
J01XD *	0.532	J01XD *	0.526	J01 *	0.523	J01XD *	0.522	J01XA *	0.525	J01DD *	0.593	J01FA *	0.28
J01 *	0.551	J01 *	0.531	J01XD *	0.525	J01 *	0.524	J01M *	0.59	J01FA *	0.644	J01DC *	0.297
J01G *	0.625	J01G *	0.572	J01M *	0.548	J01M *	0.544	J01G *	0.606	J01DH *	0.657	J01DD *	0.317
J01M *	0.625	J01DD *	0.582	J01DD *	0.567	J01DD *	0.553	J01 *	0.624	J01 *	0.666	J01CA *	0.335
J01DD *	0.63	J01M *	0.583	J01G *	0.644	J01G *	0.611	J01DH *	0.661034	J01DC *	0.673	J01M *	0.366
J01FA *	0.706	J01FA *	0.711	J01FA *	0.692	J01FA *	0.688	J01FA *	0.662	J01M *	0.772	J01CR *	0.467
J01DC *	0.716	J01DC *	0.716	J01DC *	0.73	J01DC *	0.737	J01DC *	0.704	J01CR *	0.807	J01 *	0.541

**Table 2:** Associations between 14 resistotypes and antimicrobial classes as determined in univariate regression. All associations are statistically significant (*p* < 0.05). Associations are mapped as Pearson’s correlation *R* from order of lowest risk to highest risk.

**3GCR** **EC**	***R***	**FQR EC**	***R***	**AGR** **EC**	***R***	**3XR** **EC**	***R***	**AMPR** **EC**	***R***	**VRE**	***R***	**PNS** **SP**	***R***
J01E	0.017	J01CA	−0.222	J01FF	0.463	J01E	−0.014	J01DB	−0.399	J01CE	−0.273	J01A	0.047
J01FF	0.347	J01XE	−0.025	J01FA	0.6	J01	0.417	J01A	−0.028	J01DB	−0.12	J01DC	0.296
J01	0.449	J01FF	0.304	J01DD	0.654	J01FF	0.46	J01CA	0.068	J01CA	−0.111	J01FA	0.304
J01XD	0.589	J01XD	0.59	J01M	0.657	J01FA	0.6	J01XE	0.094	J01FF	−0.014	J01XA	0.337
J01FA	0.601	J01CR	0.62	J01G	0.689	J01XD	0.611	J01FF	0.133	J01E	0.057	J01CA	0.38
J01M	0.73	J01DC	0.652	-	-	J01M	0.62	J01XD	0.386	J01XE	0.147	J01CR	0.522
J01DD	0.76	J01DD	0.666	-	-	J01DD	0.655	J01CR	0.63	J01DC	0.523	J01	0.572
-	-	J01FA	0.678	-	-	-	-	J01M	0.632	J01XD	0.596	-	-
-	-	J01M	0.81	-	-	-	-	J01FA	0.697	-	-	-	-
**3GCR KP**	***R***	**FQR KP**	***R***	**AGR** **KP**	***R***	**3XR** **KP**	***R***	**CARBR** **KP**	***R***	**MRSA**	***R***	**PNS/ER** **SP**	***R***
J01CF	−0.57	J01CF	−0.581	J01CF	−0.540	J01CF	−0.548	J01DB	−0.193	J01CF	−0.386	J01A	0.081
J01DB	−0.255	J01DB	−0.268	J01XE	0.061	J01XE	0.077	J01XE	−0.128	J01E	−0.138	J01FA	0.28
J01E	−0.071	J01A	−0.263	J01	0.523	J01	0.524	J01E	−0.028	J01CA	−0.075	J01DC	0.297
J01XE	0.068	J01E	−0.1	J01M	0.548	J01M	0.544	J01FF	0.153	J01FF	−0.016	J01CR	0.522
J01DH	0.431	J01XE	0.106	J01DD	0.567	J01DD	0.553	J01XD	0.378	J01DD	0.593	J01	0.541
J01	0.551	J01DH	0.434	J01G	0.644	J01G	0.611	J01CR	0.485	J01FA	0.644	-	-
J01DD	0.63	J01DD	0.582	J01DC	0.73	J01DC	0.737	J01DD	0.504	J01DC	0.673	-	-
J01DC	0.716	J01M	0.584	-	-	-	-	J01M	0.59	J01CR	0.807	-	-
-	-	J01DC	0.716	-	-	-	-	J01DH	0.661	-	-	-	-
-	-	-	-	-	-	-	-	J01DC	0.705	-	-	-	-

## Data Availability

All raw data are freely available in public domain. EARS-NET Antimicrobial resistance surveillance in Europe 2023–2021 data. European Centre for Disease Prevention and Control and World Health Organization; 2023. Available at https://www.ecdc.europa.eu/en/publications-data/antimicrobial-resistance-surveillance-europe-2023-2021-data. (accessed on 29 January 2025) ESAC-NET Antimicrobial consumption dashboard. Available at https://www.ecdc.europa.eu/en/antimicrobial-consumption/surveillance-and-disease-data/database. (accessed on 29 January 2025).

## Supplementary Materials

The following supporting information can be downloaded at: https://www.mdpi.com/article/10.3390/antibiotics14040399/s1; Table S1: Consumption in ddd/1000/day for each antimicrobial class in EEA countries.
