# Correction: McSorley, J.C. Analysis of ESAC-Net/EARS-Net Data from 29 EEA Countries for Spatiotemporal Associations Between Antimicrobial Use and Resistance—Implications for Antimicrobial Stewardship? *Antibiotics* 2025, *14*, 399

**DOI:** 10.3390/antibiotics15030284

**Published:** 2026-03-11

**Authors:** James C. McSorley

**Affiliations:** Department of Microbiology, Level 4, New Lister Building, Glasgow Royal Infirmary, Alexandra Parade, Glasgow, Scotland G31 2ER, UK; james.mcsorley3@nhs.scot

## Error in Figures

1. In the original publication [1], there was a mistake in “Figure 1 *E. coli* Resistance 2018 & 2021” as published. This should have been presented as a bar chart titled “*E. coli* Resistance 2017–2018 & 2020–2021 to more clearly contrast resistance in group 1 vs. group 2 countries”. The corrected Figure 1 appears below with its updated legend. 

2. In the original publication [1], there was a mistake in “Figure 2 *K. pneumoniae* Resistance 2018 & 2021” as published. This should have been presented as a bar chart titled “*K. pneumoniae* Resistance 2017–2018 & 2020–2021 to more clearly contrast resistance in group 1 vs. group 2 countries”. The corrected Figure 2 appears below with its updated legend. 

3. In the original publication [1], there was a mistake in “Figure 3 VRE & MRSA 2018 & 2021 and in Figure 4 *S. pneumoniae* Resistance 2018 & 2021” as published. These should have been presented as a bar chart titled “*S. aureus*, *E. faecium* and *S. pneumoniae* Resistance 2017–2018 & 2020–2021 to more clearly contrast resistance in group 1 vs. group 2 countries”. The corrected Figure 3 appears below with its updated legend. Note that Figures 3 and 4 from the original publication are now superseded by this single figure, affecting the numbering of subsequent figures in such a way that Figures 5, 6, 7 and 8 in the original publication are now re-numbered as Figures 4, 5, 6 and 7, respectively.

4. In the original publication [1], there was a mistake in “Figure 8. Consumption of lincosamides, nitroimidazoles, aminoglycosides, glycopeptides and carbapenems (ATCC codes J01FF, J01XD, J01G, J01XA and J01DH, respectively) in group 1 and group 2 EEA countries”. Bars representing J01G, aminoglycoside use, for seasons 2017–2018 and 2020–2021, were incorrectly ordered for group 1 vs. group 2 countries. The corrected Figure 7 appears below. 


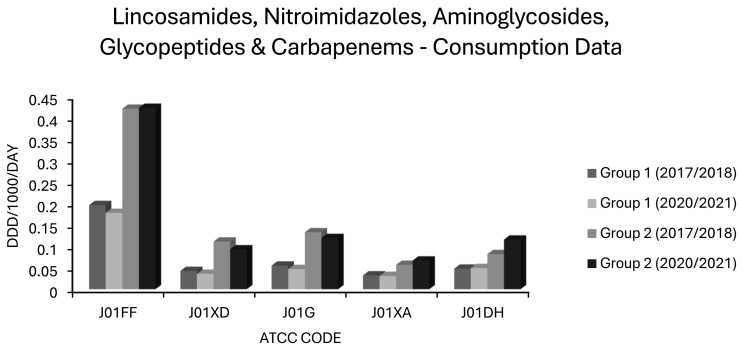


## Text Correction

There was an error in the original publication [1]. 

5. A correction has been made to “Section 2. Methods”:

In the original publication [1], the final paragraph of Section 2. Methods, read as follows: “Consumption in the 2017/2018 and 2020/2021 periods was charted for each of seven countries, with decreases in ≥2 resistotypes and increases in no more than two of the 14 resistotypes (group 1 countries) alongside consumption over the same period for each of seven countries with increases in ≥2 resistotypes (group 2 countries).” This has been changed and now reads as follows: “All VIF were ≤5. A subset of seven countries with decreases in ≥2 resistotypes and no increase in MRSA, VRE or any Gram-negative resistotype were considered to have stable or decreasing resistance and were designated as ‘group 1’ countries. Another subset of eight countries were identified as having stably high or rising resistance if they experienced increases in ≥2 resistotypes spread across at least 2 sentinel pathogens and these were designated as ‘group 2’ countries. Median levels of resistance were charted for comparison between group 1 and group 2 countries, as the percentage of isolates displaying each resistotype for the first (2017–2018) and last (2020–2021) seasons studied. Likewise, mean consumption of each antimicrobial class in group 1 and in group 2 countries for the first (2017–2018) and last (2020–2021) seasons was charted, for comparison, in ddd/1000/day.”

6. A correction has been made to “Section 3.1”. This was originally titled “Resistance in Each EEA Country” and read as follows: “Resistance levels in each EEA country were plotted graphically for the first (2018) and last (2021) of the 4 years examined for *E. coli* (Figure 1), *K. pneumoniae* (Figure 2), VRE, MRSA (Figure 3), and *S. pneumoniae* (Figure 4).” This section has been retitled as “Resistance in Group 1 and Group 2 Countries” and has been changed to read as follows: “Seven countries experienced no increase in MRSA, VRE or in any of the *E. coli* or *K. pneumoniae* resistotypes and were categorized as group 1 countries. These countries were Belgium, Denmark, Estonia, France, Ireland, Netherlands and Norway. Eight countries had increases in ≥2 resistotypes spread across at least 2 sentinel pathogens and were defined as group 2 countries. These countries were Bulgaria, Croatia, Cyprus, Greece, Hungary, Poland, Romania and Spain. Median resistance levels of each resistotype for the first (2017–2018) and final (2020–2021) seasons studied are charted below for both group 1 and group 2 countries: *E. coli* (Figure 1), *K. pneumoniae* (Figure 2), VRE, MRSA and *S. pneumoniae* (Figure 3). In general, resistance was much higher at baseline in group 2 countries with a tendency to increase overtime. Contrastingly, resistance was much lower at baseline in group 1 countries with a tendency to remain stable or decrease slowly over time.

Other countries, though included in the regression analyses, were not classified as group 1 or group 2 as they did not fit the rigid definitions used to capture dynamic changes in resistance over time. In some cases, this was because of an isolated rise in a single resistotype against a background of generally low and declining resistance. Finland and Sweden, for example, had very low resistance overall, even compared to some group 1 countries, but were not themselves assigned to group 1 due solely to isolated increases in AGR *K. pneumoniae* and MRSA, respectively. Similarly, Germany and Slovenia were excluded from group 1 because of isolated increases in VRE. Italy and Portugal had generally high resistance levels at baseline but achieved significant reductions over the analysed period for most of these resistotypes, other than VRE and CARBR *K. pneumoniae* for which they showed respective increases. Austria, Czechia and the UK were not categorised as full consumption data were not available for these countries. Note that no country achieved a sustained reduction in either of the pneumococcal resistotypes.”

7. A correction has been made to “Section 3.3”. The final sentence of this section originally read as follows: “The consideration of lower urinary breakpoints has the potential to broaden treatment options and improve antimicrobial stewardship”. This wording was incorrect and the word ‘lower’ has now been replaced with the word ‘higher’. 

8. A correction has been made to “Section 3.4”. One sentence originally read “The Gram-negative spectrum of aminopenicillins has already been much eroded by acquired resistance, but amoxicillin remains an option for the definitive treatment of infections caused by *E. coli*, *Proteus*, *Salmonella* and *H. influenzae* strains with laboratory proven sensitivity”. This has been reworded as follows, to be more specific: “The Gram-negative spectrum of aminopenicillins has already been much eroded by acquired resistance, but amoxicillin remains an option for the definitive treatment of infections caused by *E. coli*, *Proteus mirabilis*, *Salmonella* and *H. influenzae* strains with laboratory proven sensitivity”.

9. A correction has been made to “Section 3.5”. One sentence originally read “These drugs merit consideration for the management of infections caused by *E. coli*, *Klebsiella* and *Proteus* strains of established or strongly suspected susceptibility”. This has been reworded as follows, to be more specific: “These drugs merit consideration for the management of infections caused by *E. coli*, *Klebsiella* and *P. mirabilis* strains of established or strongly suspected susceptibility”. One sentence originally read “Six of seven group 2 countries had increasing J01DD use, consumption in the remaining country, Spain, was stable (Supplementary Table S1). Both second and third generation cephalosporins were strongly associated with all 14 resistotypes on univariate analysis (Table 1).” This has been reworded as follows, to be more specific: “Seven of eight group 2 countries had increasing J01DD use, consumption in the remaining country, Spain, was stable (Supplementary Table S1). Both second and third generation cephalosporins were strongly associated with all 14 resistotypes on univariate analysis (Table 1).”

10. A correction has been made to “Section 3.11”. One sentence originally read as follows: “Two of the seven group 2 countries, Bulgaria and Cyprus, increased J01M use by 0.824 and 0.928 ddd/1000/day, respectively, from already high baselines of 2.996 and 5.751 ddd/1000/day (Supplementary Table S1), with the overall effect that mean quinolone consumption in the group 2 countries was unchanged over 5 years (Figure 5).” This has been reworded as follows, to be more specific: “Two of the eight group 2 countries, Bulgaria and Cyprus, increased J01M use by 0.824 and 0.928 ddd/1000/day, respectively, from already high baselines of 2.996 and 5.751 ddd/1000/day (Supplementary Table S1), with the overall effect that mean quinolone consumption in the group 2 countries was unchanged over 5 years (Figure 4).”

11. A correction has been made to “Section 3.14”. One sentence originally read as follows: “Nitrofurantoin, though usually effective against *E. coli*, has much less consistent activity against *Klebsiella* spp. and other enterobacterial genera which may cause urinary tract infections (UTIs), especially among patients who are catheterised or have structural abnormalities of the genitourinary system”. This has been reworded as follows: “Nitrofurantoin, though usually effective against *E. coli*, has much less consistent activity against other GNB which may cause urinary tract infections (UTIs), especially among patients who are catheterised or have structural abnormalities of the genitourinary system. Susceptibility of *Klebsiella* and *Enterobacter* is variable whilst *P. aeruginosa*, *Proteeae* and *Serratia* are intrinsically resistant”.

12. A correction has been made to “Section 4”. In the original article, one paragraph read as follows: “Aminopenicillins, though having a low resistance potential overall, are associated with resistance towards amoxicillin/ampicillin in *E. coli*. Though this may not seem a significant problem given that resistance to these agents has now been widespread for decades, it should be noted that the use of these antibiotics is extremely common, as are *E. coli* UTIs. A substantive decrease in use of these agents may therefore yield a significant decline in selective pressure for resistance in *E. coli* and presumably also in other pathogens innately sensitive to these drugs, such as *Proteus* spp., *Helicobacter pylori* and *H. influenzae*. The substitution of narrower spectrum penicillins G and V as alternatives to aminopenicillins, as is the practice in Scandinavian countries, merits consideration.” This has been replaced with “Aminopenicillins, though having a low resistance potential overall, select for resistance towards amoxicillin/ampicillin, and likely also to other agents such as trimethoprim, in *E. coli*. Though this may not seem a significant problem given that resistance to these agents has now been widespread for decades, it should be noted that the use of these antibiotics is extremely common, as are *E. coli* infections of the urinary and biliary tracts, abdominal cavity and bloodstream. A substantive decrease in use of aminopenicillins may therefore yield a significant decline in selective pressure for resistance in *E. coli* and presumably also in other pathogens innately sensitive to these drugs such as *P. mirabilis*, *Helicobacter pylori* and *H. influenzae*. Where aminopenicillins are not being utilised specifically for their Gram-negative activity, preferential substitution of penicillin G or V, as is the practice in Scandinavian countries, merits consideration.”

The authors state that the scientific conclusions are unaffected. This correction was approved by the Academic Editor. The original publication has also been updated.

## Figures and Tables

**Figure 1:**
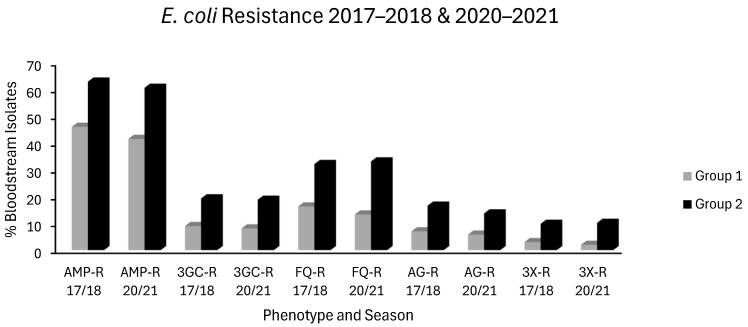
Levels of *E. coli* resistance in group 1 countries (grey) and group 2 countries (black) for the first (2017–2018) and last (2020–2021) seasons of EARS-NET data analysed chronologically.

**Figure 2:**
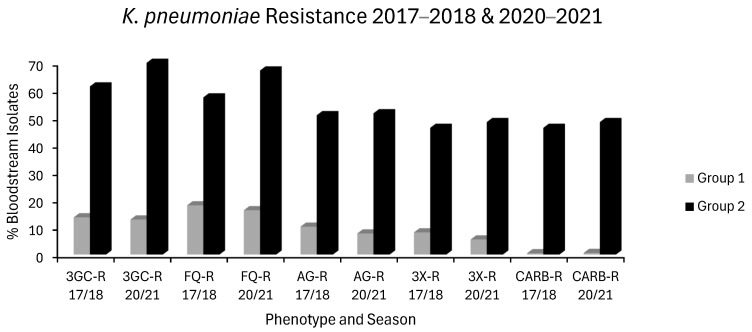
Levels of *K. pneumoniae* resistance in group 1 countries (grey) and group 2 countries (black) for the first (2017–2018) and last (2020–2021) seasons of EARS-NET data analyzed chronologically.

**Figure 3:**
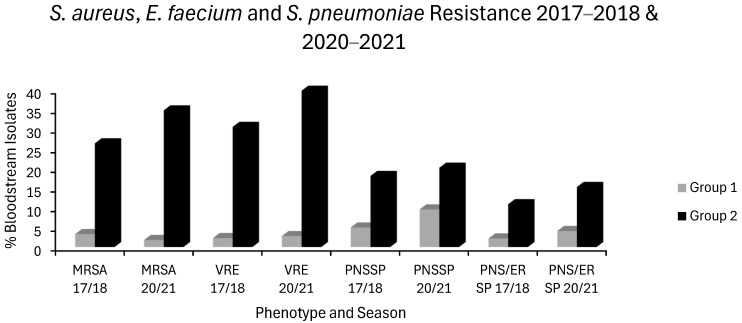
Levels of *S. aureus*, *E. faecium* and *S. pneumoniae* resistance in group 1 countries (grey) and group 2 countries (black) for the first (2017–2018) and last (2020–2021) seasons of EARS-NET data analysed chronologically.
